# Age and Behavior-Dependent Differential miRNAs Expression in the Hypopharyngeal Glands of Honeybees (*Apis mellifera* L.)

**DOI:** 10.3390/insects12090764

**Published:** 2021-08-26

**Authors:** Tengfei Shi, Yujie Zhu, Peng Liu, Liang Ye, Xingchuan Jiang, Haiqun Cao, Linsheng Yu

**Affiliations:** 1 School of Plant Protection, Anhui Agricultural University, Hefei 230036, China; Stf2020@ahau.edu.cn (T.S.); 19710072@ahau.edu.cn (P.L.); yeliang@ahau.edu.cn (L.Y.); jxc2014@ahau.edu.cn (X.J.); caohq@ahau.edu.cn (H.C.); 2 School of Animal Science and Technology, Anhui Agricultural University, Hefei 230036, China; jade@ahau.edu.cn

**Keywords:** honeybees, hypopharyngeal glands, miRNA, different developmental stages

## Abstract

**Simple Summary:**

The hypopharyngeal glands (HPGs) are a pair of aciniform glands that are located in the frontal area of the heads of worker bees (*Apis mellifera* L.) that exhibit age and behavior-dependent development. Little is known about whether/how miRNAs regulate the HPGs development. In this study, small RNA sequencing was employed to analyze the miRNA profiles of HPGs in newly-emerged bees (NEB), nurse bees (NB), and forager bees (FB). We found that there were a total of 31 known miRNAs differentially expressed among the three stages, which might have regulatory roles in the growth and development, protein synthesis, and carbohydrate and energy metabolism in the HPGs. Additionally, the downregulation of ame-miR-184-3p and ame-miR-252a-5p in nurse bees may be involved in royal jelly secretion, while the lower expression of ame-miR-11-3p and ame-miR-281-3p in forager bees are responsible for honey processing.

**Abstract:**

This study aims to investigate the expression differences of miRNAs in the hypopharyngeal glands (HPGs) of honeybees at three developmental stages and to explore their regulation functions in the HPGs development. Small RNA sequencing was employed to analyze the miRNA profiles of HPGs in newly-emerged bees (NEB), nurse bees (NB), and forager bees (FB). Results showed that a total of 153 known miRNAs were found in the three stages, and ame-miR-276-3p, ame-miR-375-3p, ame-miR-14-3p, ame-miR-275-3p, and ame-miR-3477-5p were the top five most abundant ones. Furthermore, the expression of 11 miRNAs, 17 miRNAs, and 18 miRNAs were significantly different in NB vs. FB comparison, NB vs. NEB comparison, and in FB vs. NEB comparison, respectively, of which ame-miR-184-3p and ame-miR-252a-5p were downregulated in NB compared with that in both the FB and NEB, while ame-miR-11-3p, ame-miR-281-3p, and ame-miR-31a-5p had lower expression levels in FB compared with that in both the NB and NEB. Bioinformatic analysis showed that the potential target genes of the differentially expressed miRNAs (DEMs) were mainly enriched in several key signaling pathways, including mTOR signaling pathway, MAPK signaling pathway-fly, FoxO signaling pathway, Hippo signaling pathway-fly. Overall, our study characterized the miRNA profiles in the HPGs of honeybees at three different developmental stages and provided a basis for further study of the roles of miRNAs in HPGs development.

## 1. Introduction

Honeybees (*Apis mellifera* L.) are eusocial insects, and a typical colony consists of three castes, including queen, drones, and worker bees. Amongst those, worker bees are the predominant ones that exhibit a complex age-dependent division of labor [1]. In general, within the first two weeks after emergence, the worker bees mainly perform tasks inside the hive, such as brood and queen rearing (nurse bees). After that, most of the worker bees will grow into foragers, which are responsible for collecting pollen and nectar outside the hive [2].

The structure and function of some glands, such as hypopharyngeal glands (HPGs), which are a pair of aciniform glands that are located in the frontal area of the heads of worker bees, will change accompanying the role change of bees [3,4]. In young bees within six days after eclosion, the HPGs are small and have no secretory activity [5]. However, the HPGs of 6–15-day-old nurse bees are well-developed and have strong ability to biosynthesize and secrete royal jelly (RJ), which is the primary food for queen and young larvae [6,7,8]. However, when the worker bees mature into foragers, this gland almost shrinks, but it can also produce various carbohydrate-metabolizing enzymes, such as α-glucosidase III, α-amylase, and glucose oxidase, which are used for honey processing [7,9,10,11].

Previous studies have demonstrated that age and behavior-dependent physiological and functional changes of HPGs are closely associated with differential gene expression in honeybees [12,13]. Liu et al. (2014) carried out a digital gene expression analysis of HPGs of two bee species, *A. mellifera* and *A. cerana* at three developmental stages (newly emerged bees, nurses, and foragers), and they found that there were 1482 genes in *A. mellifera* and 1313 genes in *A. cerana* differentially expressed among the three stages, respectively [14]. To identify the molecular basis of RJ production, Nie et al. (2020) recently compared the transcriptome of HPGs between nurses and foragers at the same age. Their results showed that 510 upregulated genes in nurse bees related to translation, transcription, DNA replication, and energy metabolism were strongly involved with the secretion of RJ. However, related studies on non-coding RNAs (ncRNAs) expression, such as miRNAs expression, are still elusive [15].

MicroRNA (miRNA) is a class of small (18–24 nt), single-strand, and endogenous ncRNA that plays important roles in the post-transcriptional regulation of target gene expression [16,17] related to various biological processes, such as tissue development [18]. Previous studies on honeybee miRNAs revealed their key roles in mediating diverse physiological processes, such as caste differentiation [19,20], division of labor [21,22], memory formation [23,24], queen reproductive performance [25,26], immune defense [27,28], and embryonic and midgut development [29,30]. However, little is known about whether/how miRNAs regulate the HPGs development in worker bees.

To identify the association between differential miRNAs expression and the age and behavior-dependent HPGs development, small RNA sequencing (sRNA-seq) was conducted to analyze the miRNA profiles of HPGs in worker bees at three different developmental stages (newly emerged bees, nurses, and foragers). Our results uncovered that the differentially expressed miRNAs have regulatory roles in growth and development, protein synthesis, and carbohydrate and energy metabolism in the HPGs of honeybees.

## 2. Materials and Methods

### 2.1. Honeybees and RNA Extraction

The bee samples were collected from three healthy sister-queen honeybee (*A. mellifera* L.) colonies, which were maintained at the Institute of Apicultural Research, Anhui Agricultural University, Hefei, China (GPS: 31°53′ N, 117°20′ E). The newly-emerged bees (NEB), nurse bees (NB), and forager bees (FB) were collected following the method of Nie et al. [15]. The obtained bees were first narcotized by N_2_, then we dissected the bee heads and obtained the HPGs on ice. Pools of 10–15 pairs of HPGs from each sample were prepared for total RNA extraction using trizol reagent (Invitrogen, Carlsbad, CA, USA). Additionally, 1.5% agarose gel was used to monitor RNA degradation and contamination, especially DNA contamination. We used the NanoDrop 2000 Spectrophotometer (Thermo Fisher Scientific, Wilmington, NC, USA) to measure RNA concentration and purity. The RNA Nano 6000 Assay Kit was used to assess RNA integrity on Agilent Bioanalyzer 2100 System (Agilent Technologies, Carlsbad, CA, USA).

### 2.2. Small RNA Sequencing and Analysis

A total of three micrograms of RNA per sample was used to prepare the sequencing sample. Sequencing libraries were generated using NEBNext^R^ Ultra^R^ small RNA Sample Library Prep Kit for Illumina^R^ (New England Biolabs, Beijing, China) following manufacturer’s recommendations, and index codes were added to attribute sequences to each sample. In total, nine sequencing libraries were constructed, and each group (NEB, NB, and FB) contained three libraries. The clustering of the index-coded samples was performed on a cBot Cluster Generation System using TruSeq PE Cluster Kit v4-cBot-HS (Illumina, San Diego, CA, USA) according to the manufacturer’s instructions. After cluster generation, the library preparations were sequenced on an Illumina Hiseq 4000 platform and 1 × 50 bp single-end reads were generated (Illumina). After quality control, the filter ribosomal RNA (rRNA), transfer RNA (tRNA), small nuclear RNA (snRNA), small nucleolar RNA (snoRNA), other ncRNA. and repeat sequences were removed based on high-quality clean reads mapped to Silva database, GtRNAdb database, Rfam database, and Repbase database using Bowtie software [31]. Then, the remaining reads were used to detect known miRNA by comparing with known miRNAs in *A. mellifera* from miRbase, while RNAfold software [32] was used for novel miRNA secondary structure prediction.

### 2.3. Differential Expression Analysis

The miRNA expression levels were calculated by transcript per million (TPM). Differential expression analysis between the following comparisons: (1) NB vs. FB, (2) NB vs. NEB, and (3) FB vs. NEB, were calculated by the DESeq R package [33]. The miRNAs with a *p*-value < 0.01 and |log2-fold change| > 1 were assigned as significantly differentially expressed. The 3′-untranslated region (3′-UTR) sequences from the *A. mellifera* genome were used to predict the target genes of the differentially expressed miRNAs (DEMs) with miRanda [34] and TargetScan [35] software. The predicted target genes with miranda_energy < −10 or TargetScan_score ≥ 50 were obtained. We extracted the overlapping target genes identified using the two pieces of software. Gene ontology (GO) enrichment analysis of the predicted target genes was implemented by the topGO R package [36], while we used KOBAS software [37] to test the statistical enrichment of predicted target genes in Kyoto Encyclopedia of Genes and Genomes (KEGG) pathways.

### 2.4. Validation of Known DEMs by qPCR

Ten of the differentially expressed miRNAs (ame-miR-124-3p, ame-miR-184-3p, ame-miR-210-3p, ame-miR-3049-5p, ame-miR-31a-5p, ame-miR-932-5p, ame-miR-993-3p, ame-miR-11-3p, ame-miR-8-3p, and ame-miR-3477-5p) were randomly selected for qPCR validation. One microgram of total RNA was input to obtain the cDNA using the miRcute Plus miRNA First-Strand cDNA Kit (Tiangen, Beijing, China) on a PCR system (Bioer, Hangzhou, China), while qPCR was performed with miRcute Plus miRNA qPCR Kit (SYBR Green) (Tiangen) on a StepOnePlus Real-Time PCR System (Thermo Fisher Scientific). U6 was used as reference gene, and the detailed information of primers used in the present study are listed in Table 1. The relative expression levels of miRNAs were calculated by 2^−ΔCT^ method [38]. The Student’s *t*-test was used to analyze the differences in miRNAs expression between each of the pairwise comparisons.

## 3. Results

### 3.1. Small RNA Sequencing Analysis

**Figure 1:**
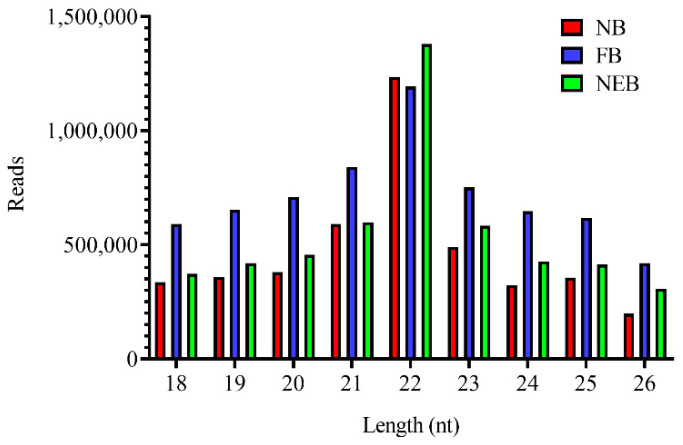
Distribution of the lengths of the small RNA reads in the nurse bee (NB), forager bee (FB), and newly-emerged bee (NEB) libraries.

Using the Illumina Hiseq 4000 platform, nine sRNA libraries were subjected to deep sRNA sequencing. After removing the useless sequences, 3.27 to 6.85 million clean reads were obtained (Table S1). The length distribution of the small RNAs for all three groups peaked at 22 nt, with 16.87% to 31.36% of total clean reads (Figure 1).

After mapping to Silva database, GtRNAdb database, Rfam database, and Repbase database, the clean reads were divided into different categories, and 60.71–72.30% belonged to unique rRNAs, 5.85–15.54% were unique tRNAs, 0.87–2.83% were unique snRNAs, and 0.50–1.47% were unique snoRNAs. On the other hand, the remaining 17.56–20.97% unannotated reads containing miRNAs were used to identify the known miRNAs and novel miRNAs.

### 3.2. Identification of Known and Novel miRNAs

**Figure 2:**
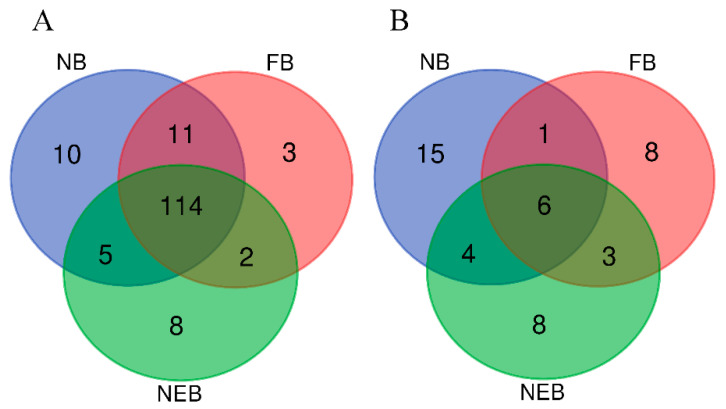
Distribution of known miRNAs (**A**) and novel miRNAs (**B**) in the nurse bee (NB), forager bee (FB), and newly-emerged bee (NEB) libraries.

We compared the unannotated sequences with known mature miRNAs of *A. mellifera* in the miRBase database to identify the known miRNAs. In total, 140, 130, and 129 known miRNAs were found in NB HPGs, FB HPGs, and NEB HPGs, respectively. Among them, 114 were shared between three groups (Figure 2A, Table S2). Ten miRNAs with the highest expression were selected and listed in Table 2, and five of them (ame-miR-276-3p, ame-miR-375-3p, ame-miR-14-3p, ame-miR-275-3p, and ame-miR-3477-5p) were expressed in all groups. Remarkably, ame-miR-276-3p, ame-miR-275-3p, and ame-miR-14-3p were the top three most abundant miRNAs with 322589 reads, 122003 reads, and 72753 reads, respectively.

In total, 26, 18, and 21 novel miRNAs were predicted in NB HPGs, FB HPGs, and NEB HPGs, respectively. Among them, six novel miRNAs were shared in the three groups, while seven, nine, and ten novel miRNAs were common in NB vs. FB, FB vs. NEB, and NB vs. NEB comparisons, respectively. Otherwise, fifteen, eight, and eight novel miRNAs were only expressed in NB HPGs, FB HPGs, and NEB HPGs, respectively (Figure 2B, Table S3).

### 3.3. Differential Expression Analysis of miRNAs

**Figure 3:**
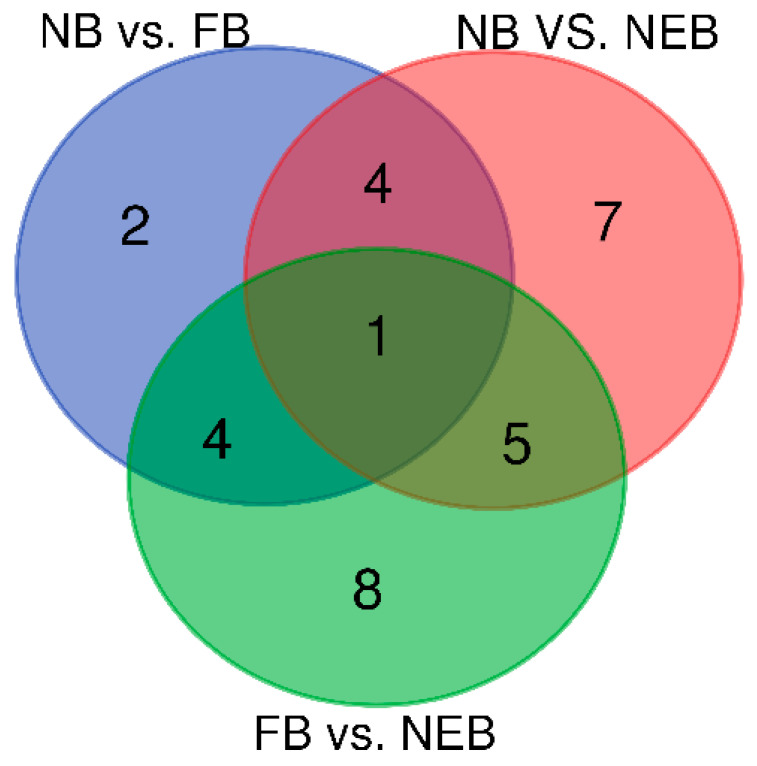
Differentially expressed miRNAs among nurse bee (NB), forager bee (FB), and newly-emerged bee (NEB) libraries.

As shown in Table 3, there were 31 miRNAs differentially expressed among the NEB, NB, and FB libraries (*p* < 0.01 and |log2-fold change| > 1). Among these, 11 miRNAs exhibited differential expression in the NB vs. FB comparison (five upregulated and six downregulated), 17 miRNAs in NB vs. NEB comparison (five upregulated and twelve downregulated), and 18 miRNAs in FB vs. NEB comparison (six upregulated and twelve downregulated). In addition, one miRNA, ame-miR-31a-5p, was common in the three comparisons; five miRNAs were common between NB vs. FB and NB vs. NEB comparisons, five miRNAs between NB vs. NEB and FB vs. NEB comparisons, and six miRNAs between FB vs. NEB and NB vs. NEB comparisons (Figure 3, Table 3). Some differentially expressed miRNAs (DEMs) called our attention: ame-miR-3477-5p and ame-miR-281-3p had higher expression in NB than in both FB and NEB, while ame-miR-184-3p and ame-miR-252a-5p showed the contrary expression tendency (Table 4); ame-miR-3791-3p and ame-miR-210-3p were upregulated in FB compared with that both in NB and NEB, while ame-miR-11-3p, ame-miR-283-5p, and ame-miR-31a-5p were downregulated (Table 4).

### 3.4. Prediction of the Target Genes of DEMs and Enrichment Analysis

**Figure 4:**
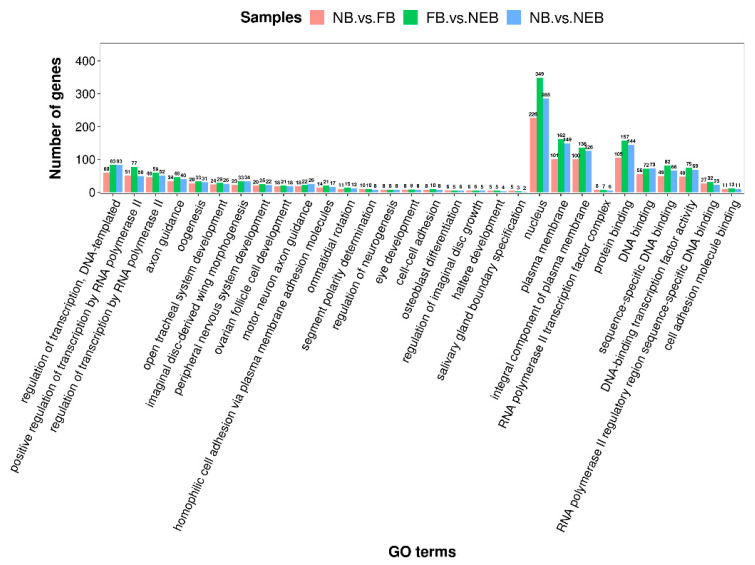
Gene ontology (GO) enrichment analysis of predicted target genes of the differentially expressed miRNAs identified in the NB vs. FB comparison, NB vs. NEB comparison, and FB vs. NEB comparison. NB: nurse bee, FB: forager bee, NEB: newly-emerged bee.

**Figure 5:**
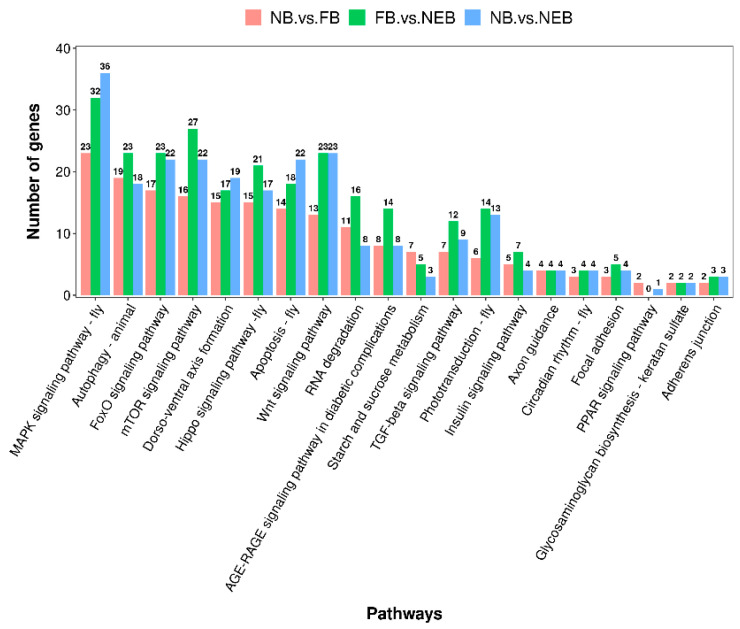
KEGG pathways analysis of predicted target genes of the differentially expressed miRNAs identified in the NB vs. FB comparison, NB vs. NEB comparison, and FB vs. NEB comparison. NB: nurse bee, FB: forager bee, NEB: newly-emerged bee.

Using miRanda and TargetScan softwares, we predicted the target genes of the 31 DEMs among the NEB, NB, and FB libraries. In total, 1180, 1531, and 1868 candidate target genes were obtained for 11 DEMs confirmed in the NB vs. FB comparison (Table S4), 17 DEMs confirmed in the NB vs. NEB comparison (Table S5), and 18 DEMs confirmed in the FB vs. NEB comparison (Table S6), respectively. The predicted target genes of these DEMs obtained in the three comparisons were enriched in thousands of GO terms. Nucleus, plasma membrane, protein binding, integral component of plasma membrane, and regulation of transcription, DNA-templated were the top five most enriched ones (Figure 4, Tables S7–S9). The predicted target genes were mapped to 217 KEGG pathways, and the mTOR signaling pathway, MAPK signaling pathway-fly, FoxO signaling pathway, Hippo signaling pathway-fly, and Autophagy-animal were the top five most abundant ones (Figure 5, Tables S10–S12).

### 3.5. qPCR Analysis 

**Figure 6:**
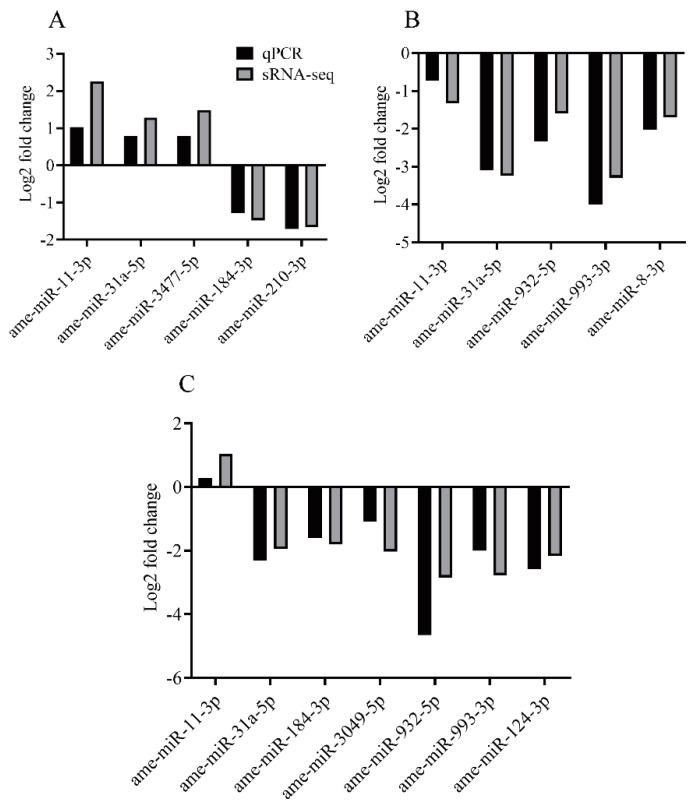
Validation of differentially expressed miRNAs confirmed in the NB vs. FB comparison (**A**), the FB vs. NEB comparison (**B**), and the NB vs. NEB comparison (**C**) by qPCR analysis. NB: nurse bee, FB: forager bee, NEB: newly-emerged bee.

Five DEMs confirmed in the NB vs. FB comparison, six DEMs identified in the NB vs. NEB comparison, and five DEMs obtained in the FB vs. NEB comparison were validated by qPCR assay. As shown in Figure 6, the miRNA expression patterns exhibited a similar trend between the qPCR results and the sRNA-seq data. In the NB vs. FB comparison, ame-miR-11-3p, ame-miR-31a-5p, and ame-miR-3477-5p were upregulated, while ame-miR-184-3p and ame-miR-210-3p were downregulated. In the NB vs. NEB comparison, ame-miR-11-3p was upregulated, and ame-miR-184-3p, ame-miR-124-3p, ame-miR-3049-5p, ame-miR-31a-5p, ame-miR-932-5p, and ame-miR-993-3p were downregulated. In the FB vs. NEB comparison, ame-miR-11-3p, ame-miR-31a-5p, ame-miR-8-3p, ame-miR-932-5p, and ame-miR-993-3p were all downregulated. 

## 4. Discussion

In this study, sRNA-seq was employed to explore the miRNA profiles in the hypopharyngeal glands of honeybees at three developmental stages (newly-emerged bees, nurse bees, and forager bees). In total, 153 known miRNAs were obtained, and 114 of them were shared in the three stages. Differential expression analysis showed that 11, 17, and 18 miRNAs were significantly differentially expressed between NB vs. FB comparison, FB vs. NEB comparison, and NB vs. NEB comparison, respectively (Figure 3, Table 3). Validation of ten DEMs using qPCR indicated that the results of qPCR and sRNA-seq showed a similar trend, suggesting the reliability of sRNA-seq data [39]. The potential target genes of DEMs were also predicted, which mainly participated in diverse signaling pathways, including mTOR signaling pathway, MAPK signaling pathway-fly, FoxO signaling pathway, and Hippo signaling pathway-fly (Figure 5, Tables S10–S12), which might play important roles in regulating HPGs development in honeybees.

Among the 153 known miRNAs obtained in the present study, ame-miR-276-3p, ame-miR-375-3p, ame-miR-14-3p, ame-miR-275-3p, and ame-miR-3477-5p were the top five most abundant miRNAs that commonly existed in NB, FB, and NEB libraries (Table 2). A recent study demonstrated that ame-miR-14 was enriched in the queen ovary and played an important role in regulating egg-laying via modulating *ecdysone receptor* in honeybee queens [26]. The miRNA ame-mir-276 is highly expressed in the small-type Kenyon cells of the mushroom bodies and is involved with the development of related neural function in honeybees [40]. Therefore, the highly expressed miRNAs in our study might play indispensable roles in the HPGs development in honeybees. Furthermore, our study also found that the expression level of ame-miR-3477-5p exhibited significant differences in NB vs. FB comparison or in NB vs. NEB comparison, and ame-miR-276-3p had higher expression in NEB than that in NB. Previous studies reported that there were striking differences in the brain or head miRNAs expression between nurse and forager [21,22,41,42]. Hence, development stages have obvious impacts on the expression levels of miRNAs in honeybees [43,44].

There were a total of 31 differentially expressed miRNAs confirmed among the three different developmental stages, and several of them interested us enormously. For instance, ame-miR-184-3p and ame-miR-252a-5p had lower expression in NB than that in both the FB and NEB (Table 3). The miRNA ame-miR-184-3p is found to be involved in royal jelly secretion by enhancing the expression of the target genes, *insulin-like receptor* and *cyclin dependent kinase 12*, and inhibition of ame-miR-184-3p expression also can activate the mTOR signaling pathway [45]. Accordingly, many potential target genes of ame-miR-184-3p obtained in the present study were enriched for this key signaling pathway (Table 4). In addition, many predicted target genes of ame-miR-184-3p and ame-miR-252a-5p were also responsible for protein synthesis and energy metabolism (Table 4), which is similar to a previous study declaring that the upregulated genes in the HPGs of NB were notably enriched in ribosome and aminoacyl-tRNA biosynthesis [15]. It is well known that the HPGs of nurse bees have strong activity to secrete RJ protein [13,46], and HPGs of high-RJ-producing nurse bees exhibit stronger energy replenishment than those of Italian bees [47]. Hence, the lower expression of ame-miR-184-3p and ame-miR-252a-5p in nurse bees was closely associated with RJ secretion.

On the other hand, three miRNAs (ame-miR-11-3p, ame-miR-281-3p, and ame-miR-31a-5p) showed lower expression levels in FB compared with that in both the NB and NEB (Table 3). Similarly, a previous study found that ame-miR-31a had higher expression level in brains of NB than in brains of FB in both the typical colonies and single-cohort colonies, which was considered as an important regulator of the behavioral transition in worker bees [48]. The miRNAs can regulate various biological processes by suppressing their target gene expression [49]. Our present study found that several potential targets of ame-miR-11-3p (glucosidase 2 subunit beta and hexosaminidase D) and ame-miR-281-3p (myogenesis-regulating glycosidase) encoded the enzymes involved in carbohydrate digestion. Glucosidase can catalyze polysaccharides into glucose [50]. Hexosaminidase D participated in glycan degradation, and myogenesis-regulating glycosidase was responsible for starch and sucrose metabolism (Table 4). The downregulation of ame-miR-11-3p and ame-miR-281-3p in HPGs of FB are likely involved with the conversion of nectar into honey [10,51]. Otherwise, some candidate target genes of ame-miR-11-3p, ame-miR-31a-5p, and ame-miR-281-3p were enriched in apoptosis-fly pathway (Table 4). Previous studies demonstrated that the HPGs begin to degenerate in worker bees more than 15 days old (forager bees) [52,53]. 

Bioinformatic analysis showed that mTOR signaling pathway, MAPK signaling pathway-fly, FoxO signaling pathway, Hippo signaling pathway-fly, and Autophagy-animal were the top five most enriched pathways in the potential target genes of the DEMs in the three comparisons: in NB vs. FB comparison, in NB vs. NEB comparison, and in FB vs. NEB comparison, which suggested that these pathways might play critical roles in hypopharyngeal glands development in honeybees. Among these, mTOR signaling has been implicated in the modulation of ribosome oogenesis and protein synthesis in cells [54,55]. Hippo signaling pathway is involved with organ size control and tissue regeneration via cell proliferation and apoptosis [56,57]. FoxO signaling pathway is responsible for carbohydrate and energy metabolism [58,59]. MAPK signaling pathway plays a significant role in regulating division of labor, caste differentiation, and queen development in honeybees [60,61,62]. These findings indicate that the DEMs might have regulatory roles in the growth and development, protein synthesis, and carbohydrate and energy metabolism in the HPGs of honeybees.

## 5. Conclusions

We first compared the miRNA profiles of hypopharyngeal glands in honeybees at different developmental stages (newly-emerged bees, nurse bees, and forager bees). Bioinformatic analysis showed that the differentially expressed miRNAs were involved in important biological processes related to growth and development, protein synthesis, and carbohydrate and energy metabolism in the hypopharyngeal glands. Additionally, we found that the downregulation of ame-miR-184-3p and ame-miR-252a-5p in nurse bees may be involved in royal jelly secretion, while the lower expression of ame-miR-11-3p and ame-miR-281-3p in forager bees are responsible for honey processing. These findings should provide a basis for further study of the roles of miRNAs in hypopharyngeal glands development.

## Figures and Tables

**Table 1:** Primers used in the present study.

miR-Name	Primer Sequences (5′ to 3′)	Amplification Efficiencies
ame-miR-124-3p	F: TAAGGCACGCGGTGAATGC	0.98
ame-miR-184-3p	F: CCTTATCATTCTCCTGTCCGGT	0.97
ame-miR-210-3p	F: GCGTTGTGCGTGTGACA	0.97
ame-miR-3049-5p	F: CGTCGGGAAGGTAGTTGC	0.95
ame-miR-31a-5p	F: GGCAAGATGTCGGCATA	0.93
ame-miR-932-5p	F: CCGTCAATTCCGTAGTGCATTGCAG	0.93
ame-miR-993-3p	F: GCGAAGCTCGTCTCTACAGGTATCT	0.94
ame-miR-11-3p	F: CGCATCACAGGCAGAGTTCTAGTT	0.96
ame-miR-8-3p	F: CGCGCGTAATACTGTCAGGTAAAGATG	1.05
ame-miR-3477-5p	F: CGCTAATCTCATGCGGTAACTGTGAG	0.97
U6 (Reference)	F: GTTAGGCTTTGACGATTTCG	1.02
	R: GGCATTTCTCCACCAGGTA	

**Table 2:** Ten highest expressed miRNAs in the hypopharyngeal glands of nurse bee, forager bee, and newly-emerged bee.

Rank	Nurse Bee		Forager Bee		Newly-Emerged Bee	
	miR-Name	Reads Count	miR-Name	Reads Count	miR-Name	Reads Count
1	ame-miR-276-3p	242,234	ame-miR-276-3p	238,540	ame-miR-276-3p	486,993
2	ame-miR-375-3p	221,939	ame-miR-375-3p	68,526	ame-miR-375-3p	75,545
3	ame-miR-14-3p	104,814	ame-miR-14-3p	42,487	ame-miR-14-3p	70,958
4	ame-miR-3477-5p	86,004	ame-miR-317-3p	28,086	ame-miR-3477-5p	38,165
5	ame-miR-317-3p	62,651	ame-miR-3477-5p	22,349	ame-miR-275-3p	33,428
6	ame-miR-8-3p	52,599	ame-miR-275-3p	18,213	ame-miR-8-3p	17,168
7	ame-miR-306-5p	38,992	ame-miR-2796-3p	16,675	ame-miR-2796-3p	16,052
8	ame-miR-275-3p	38,214	ame-miR-100-5p	13,408	ame-miR-263a-5p	14,172
9	ame-miR-12-5p	27,484	ame-miR-34-5p	7870	ame-miR-100-5p	11,417
10	ame-miR-11-3p	14,083	ame-miR-252a-5p	7759	ame-miR-252a-5p	11,363

**Table 3:** Differentially expressed miRNAs among nurse bee, forager bee, and newly-emerged bee.

DEMs	NB vs. FB			NB vs. NEB			FB vs. NEB		
	Log2 FC	*p*-Values	Regulated	Log2 FC	*p*-Values	Regulated	Log2 FC	*p*-Values	Regulated
ame-miR-11-3p	2.25	7.18 × 10^−6^	up	-	-	-	−1.33	7.62 × 10^−4^	down
ame-miR-281-3p	1.23	1.90 × 10^−3^	up	1.7	3.18 × 10^−3^	up	-	-	-
ame-miR-283-5p	2.11	2.05 × 10^−3^	up	-	-	-	−1.81	6.04 × 10^−3^	down
ame-miR-31a-5p	1.28	9.45 × 10^−3^	up	−1.95	2.10 × 10^−3^	down	−3.23	2.82 × 10^−3^	down
ame-miR-3477-5p	1.49	8.72 × 10^−4^	up	1.03	1.12 × 10^−4^	up	-	-	-
ame-miR-184-3p	−1.48	2.45 × 10^−3^	down	−1.81	6.45 × 10^−4^	down	-	-	-
ame-miR-210-3p	−1.67	8.23 × 10^−3^	down	-	-	-	1.43	8.56 × 10^−3^	up
ame-miR-252a-5p	−2.24	8.64 × 10^−3^	down	−2.74	4.80 × 10^−3^	down	-	-	-
ame-miR-2788-3p	−1.57	7.12 × 10^−4^	down	-	-	-	-	-	-
ame-miR-3791-3p	−1.66	7.49 × 10^−3^	down	-	-	-	4.91	8.03 × 10^−4^	up
ame-miR-6047a-5p	−1.15	5.72 × 10^−3^	down	-	-	-	3.36	2.22 × 10^−3^	up
ame-miR-279c-3p	-	-	-	1.38	1.26 × 10^−3^	up	-	-	-
ame-miR-6040-3p	-	-	-	3.63	9.35 × 10^−3^	up	-	-	-
ame-miR-6044-5p	-	-	-	1.7	2.27 × 10^−4^	up	-	-	-
ame-miR-124-3p	-	-	-	−2.17	3.82 × 10^−4^	down	-	-	-
ame-miR-125-5p	-	-	-	−1.33	3.02 × 10^−3^	down	-	-	-
ame-miR-276-3p	-	-	-	−1.16	7.17 × 10^−3^	down	-	-	-
ame-miR-3049-5p	-	-	-	−2.03	4.30 × 10^−3^	down	-	-	-
ame-miR-3785-3p	-	-	-	−2.13	1.14 × 10^−5^	down	−1.54	3.05 × 10^−3^	down
ame-miR-92b-3p	-	-	-	−2.36	3.75 × 10^−4^	down	−1.56	2.18 × 10^−3^	down
ame-miR-92c-3p	-	-	-	−1.41	8.15 × 10^−3^	down	-	-	
ame-miR-932-5p	-	-	-	−2.85	1.80 × 10^−3^	down	−1.59	3.34 × 10^−3^	down
ame-miR-993-3p	-	-	-	−2.77	6.31 × 10^−3^	down	−3.29	5.74 × 10^−3^	down
ame-miR-263b-5p	-	-	-	-	-	-	2.32	3.44 × 10^−3^	up
ame-miR-3770-5p	-	-	-	-	-	-	-	3.46 × 10^−3^	up
ame-miR-6052-5p	-	-	-	-	-	-	1.55	2.58 × 10^−3^	up
ame-miR-12-5p	-	-	-	-	-	-	−1.12	3.28 × 10^−3^	down
ame-miR-305-5p	-	-	-	-	-	-	−2.08	2.54 × 10^−5^	down
ame-miR-3715-5p	-	-	-	-	-	-	-	2.75 × 10^−3^	down
ame-miR-79-3p	-	-	-	-	-	-	−1.23	2.34 × 10^−3^	down
ame-miR-8-3p	-	-	-	-	-	-	−1.7	8.68 × 10^−5^	down

Note: DEMs: differentially expressed miRNAs; NB: nurse bee; FB: forager bee; NEB: newly-emerged bee; Log2 FC: Log2-fold change.

**Table 4:** The notable potential target genes of five selected differentially expressed miRNAs.

miR-Name	Potential Target Gene Symbol	Target Gene Description	KEGG Pathways	Regulation of miRNAs
ame-miR-252a-5p	STT3B	STT3, subunit of the oligosaccharyltransferase complex, homolog B [*Apis mellifera*]	ko04141 (Protein processing in endoplasmic reticulum)	Downregulated in hypopharyengeal glands of nurse
	GstZ1	glutathione S-transferase Z1 [*Apis mellifera*]	ko00350 (Tyrosine metabolism)	
	LOC551016	midasin [*Apis mellifera*]	ko03008 (Ribosome biogenesis in eukaryotes)	
	LOC413650	nuclear RNA export factor 1 [*Apis mellifera*]	ko03008 (Ribosome biogenesis in eukaryotes)	
	SdhA	succinate dehydrogenase A [*Apis mellifera*]	ko00020 (Citrate cycle); ko00190 (Oxidative phosphorylation)	
	LOC113218834	cytochrome b-c1 complex subunit 8-like [*Apis mellifera*]	ko00190 (Oxidative phosphorylation)	
	LOC724652	uncharacterized protein LOC724652 [*Apis mellifera*]	ko00020 (Citrate cycle); ko00190 (Oxidative phosphorylation)	
	Ilp-2	insulin-like peptide 2 [*Apis mellifera*]	ko04150 (mTOR signaling pathway)	
	Pten	phosphatase and tensin-like [*Apis mellifera*]	ko04150 (mTOR signaling pathway)	
	Dad	daughters against dpp [*Apis mellifera*]	ko04350 (TGF-beta signaling pathway)	
ame-miR-184-3p	LOC410870	proton channel OtopLc [*Apis mellifera*]	ko03010 (Ribosome)	
	InRS	insulin receptor substrate 1-B [*Apis mellifera*]	ko04150 (mTOR signaling pathway)	
	LOC551830	ral GTPase-activating protein subunit alpha-1 [*Apis mellifera*]	ko04150 (mTOR signaling pathway); ko04115 (p53 signaling pathway); ko04910 (Insulin signaling pathway)	
	LOC100577028	insulin-like growth factor I [*Apis mellifera*]	ko04150 (mTOR signaling pathway)	
ame-miR-11-3p	LOC410744	glucose dehydrogenase [FAD, quinone] [*Apis mellifera*]	-	Downregulated in hypopharyengeal glands of forager
	LOC413098	glucose dehydrogenase [FAD, quinone] [*Apis mellifera*]	-	
	LOC552747	glucosidase 2 subunit beta [*Apis mellifera*]	-	
	LOC551303	hexosaminidase D [*Apis mellifera*]	ko00511 (Other glycan degradation)	
	LOC412161	broad-complex core protein isoforms 1/2/3/4/5 isoform X1 [*Apis mellifera*]	ko04214 (Apoptosis-fly)	
	Mblk-1	transcription factor mblk-1-like [*Apis mellifera*]	ko04214 (Apoptosis-fly)	
	E74	ecdysteroid-regulated gene E74 [*Apis mellifera*]	ko04214 (Apoptosis-fly)	
	LOC100577393	epidermal growth factor receptor-like [*Apis mellifera*]	ko04214 (Apoptosis-fly)	
ame-miR-283-5p	LOC726210	myogenesis-regulating glycosidase [*Apis mellifera*]	ko00052 (Galactose metabolism); ko00500 (Starch and sucrose metabolism)	
	LOC726547	protein abrupt [*Apis mellifera*]	ko04214 (Apoptosis-fly)	
ame-miR-31a-5p	LOC726547	protein abrupt [*Apis mellifera*]	ko04214 (Apoptosis-fly)	

## Data Availability

Upon acceptance, the data used in this study will be available in the Supplementary Data File. The sequencing data are available in the SRA database (PRJNA754270) of the NCBI system.

## Supplementary Materials

The following are available online at https://www.mdpi.com/article/10.3390/insects12090764/s1, Table S1: Details of the sRNA-seq data; Table S2: The known miRNAs detected in NB, FB, and NEB libraries; Table S3: The novel miRNAs detected in NB, FB, and NEB libraries; Table S4: Predicted target genes of differentially expressed miRNAs confirmed in the NB vs. FB comparison; Table S5: Predicted target genes of differentially expressed miRNAs confirmed in the NB vs. NEB comparison; Table S6: Predicted target genes of differentially expressed miRNAs confirmed in the FB vs. NEB comparison; Table S7: GO enrichment analysis of predicted target genes of differentially expressed miRNAs confirmed in the NB vs. FB comparison; Table S8: GO enrichment analysis of predicted target genes of differentially expressed miRNAs confirmed in the NB vs. NEB comparison; Table S9: GO enrichment analysis of predicted target genes of differentially expressed miRNAs confirmed in the FB vs. NEB comparison; Table S10: KEGG pathways analysis of predicted target genes of differentially expressed miRNAs confirmed in the NB vs. FB comparison; Table S11: KEGG pathways analysis of predicted target genes of differentially expressed miRNAs confirmed in the NB vs. NEB comparison; Table S12: KEGG pathways analysis of predicted target genes of differentially expressed miRNAs confirmed in the FB vs. NEB comparison.
